# Neuronal Activity in the Hibernating Brain

**DOI:** 10.3389/fnana.2019.00071

**Published:** 2019-07-09

**Authors:** Mandy Sonntag, Thomas Arendt

**Affiliations:** Paul-Flechsig-Institute of Brain Research, Medical Faculty, University of Leipzig, Leipzig, Germany

**Keywords:** hibernation, hypothermia, neuronal activity, electrophysiology, c-Fos, firing rate homeostasis

## Abstract

Hibernation is a natural phenomenon in many species which helps them to survive under extreme ambient conditions, such as cold temperatures and reduced availability of food in the winter months. It is characterized by a dramatic and regulated drop of body temperature, which in some cases can be near 0°C. Additionally, neural control of hibernation is maintained over all phases of a hibernation bout, including entrance into, during and arousal from torpor, despite a marked decrease in overall neural activity in torpor. In the present review, we provide an overview on what we know about neuronal activity in the hibernating brain focusing on cold-induced adaptations. We discuss pioneer and more recent *in vitro* and *in vivo* electrophysiological data and molecular analyses of activity markers which strikingly contributed to our understanding of the brain’s sensitivity to dramatic changes in temperature across the hibernation cycle. Neuronal activity is markedly reduced with decreasing body temperature, and many neurons may fire infrequently in torpor at low brain temperatures. Still, there is convincing evidence that specific regions maintain their ability to generate action potentials in deep torpor, at least in response to adequate stimuli. Those regions include the peripheral system and primary central regions. However, further experiments on neuronal activity are needed to more precisely determine temperature effects on neuronal activity in specific cell types and specific brain nuclei.

## Introduction

Hibernation is an adaptation to extreme environmental conditions, including cold temperatures and reduced food availability. In small mammalian hibernators, it is characterized by reduced metabolism, by severely decreased heart and respiratory rates and by dramatic lowering of body temperatures to few degrees above ambient temperature (Nedergaard and Cannon, 1990; Nurnberger, 1995). Also, the brain temperature drops to 2–3°C above ambient temperature (Strumwasser, 1959). Still, hibernators are able to arouse from the state of torpor without any damage. The mechanisms underlying the entrance into and arousal from torpor state are not fully clear. However, the central nervous system (CNS) is supposed to be a fundamental key player in its regulation (Heller, 1979). This presumes that brain function is maintained under extreme hypothermia during deep torpor, though the membrane potential of neurons, the properties of ion channels and receptors, the efficiency of synaptic transmission and the conduction velocity of neuronal activity are strongly dependent on temperature, and significant, functional disruptions are already observed few degrees below physiological tissue temperature (Hodgkin and Huxley, 1952; Westerfield et al., 1978; Schiff and Somjen, 1985; Kufel and Wojcik, 2018). How does the brain of a hibernator deal with the low levels of brain temperatures? What changes in neuronal signal processing occur when the brain temperature drops to values near or below freezing point? Various approaches have been used to unravel the physiology of neurons and synapses in the hibernating brain. We presently provide a compact overview on *in vivo* and *in vitro* electrophysiological data as well as histochemical, molecular and biochemical results comprising the evaluation of activity markers. We refer to both earlier and more recent data, thereby focusing on the temperature-dependency of neuronal activity during hibernation. Structural plasticity which underlies functional changes (e.g., synapse and spine degradation, protein synthesis) is not considered here, but was intensively discussed in two recent reviews (Arendt and Bullmann, 2013; Horowitz and Horwitz, 2019).

## Electrophysiological Characterization of Neuronal Activity in the Hibernating Brain *in vivo*

### Activity of Single-Units and Neuronal Populations

Brain activity was reported to dramatically change during hibernation. Electroencephalograms (EEGs) that serve as a measure of synchronous firing of a population of neurons were nearly isoelectric (i.e., no significant changes in voltages) in hibernating ground squirrels and hamsters at lowest body temperature during torpor state (Chatfield et al., 1951; Mihailovic, 1972). Single-unit recordings obtained by chronically implanted electrodes in the lateral posterior thalamus and posterior thalamic nuclear group of ground squirrels confirmed these observations: firing rates of spontaneous neuronal activity were systematically reduced with decreasing body temperature, and neurons eventually stopped firing at body temperatures of 15–18°C, remained silent for 10–28 h (deep torpor), and only began firing again when body temperature increased (Krilowicz et al., 1988). Along with the reduction in firing rate, the waveform of the action potential dramatically changed with amplitude becoming smaller and spike width being increased at lower body temperatures (Krilowicz et al., 1989). These data imply that neuronal activity in hibernating animals is highly dependent on body and brain temperature.

### Activity of Sensory Systems and Arousal

Although electrical activity is greatly reduced and almost silent at the lowest body temperature in torpor, this does not mean that information processing is suspended. Rather, sensory stimulation was shown to elicit specific responses in the CNS, even at very low body temperatures (<10°C). Peripheral stimulation of hibernating hamsters led to electrical responses in the cerebral cortex at a body temperature of 9.1°C (Chatfield et al., 1951). Similar observations were made in the ground squirrel where touching the torpid animal with a glass rod induced activity in the brainstem reticular formation, partly followed by arousal of the animals (Strumwasser, 1959). Also, acoustic stimulation of ground squirrels was reported to evoke a behavioral response or even a complete arousal (Strumwasser, 1959). Auditory brainstem recordings (ABRs) in ground squirrels during arousal revealed that acoustic stimulation indeed elicits stimulus-driven responses even at body temperatures as low as 9.8°C (Hamill et al., 1989). In the woodchuck, auditory stimulation resulted in specific ABR responses, although only waves 1 and 2 (representing the 8th nerve and the cochlear nucleus) were detectable at lowest body temperature (Katbamna et al., 1992). These data imply that peripheral nerves and primary central brain regions might be more resistant to hypothermia-induced activity changes compared to higher central brain regions.

### Regional Pattern of Activity Changes

Reduction of brain activity as the animal enters torpor follows a regional pattern, with EEG activity being reduced first in the neocortex and then in the reticular formation, followed by thalamocortical structures and the limbic system, with activity ceasing last in the hippocampus, if at all. During arousal, activity reappears and increases in reverse order: i.e., first in the limbic system (hippocampus) and then in the hypothalamus, thalamus, reticular formation, and cortex (South et al., 1968; Mihailovic, 1972; Heller, 1979). Notably, the neocortex is the first structure where activity is depressed during entrance into torpor and the last to regain activity on arousal, a finding that has focused studies on information processing during torpor to subcortical brain regions.

**Figure 1:**
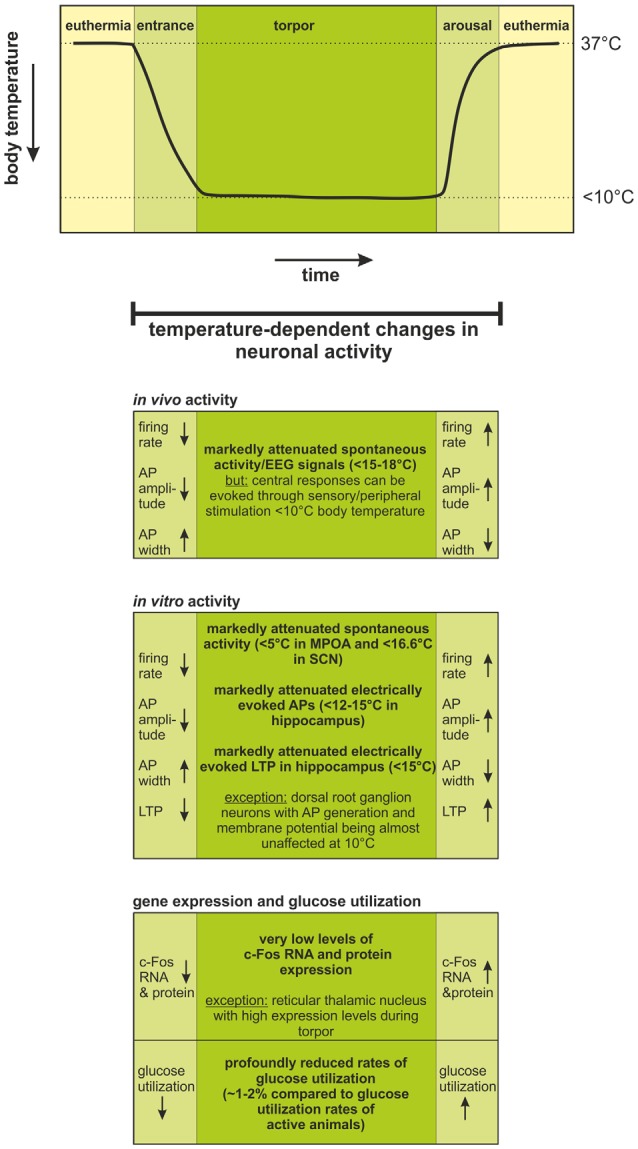
Temperature-dependent changes in neuronal activity during the hibernation cycle. Changes in body temperature during one torpor-arousal cycle are schematically illustrated in the upper graph.During torpor, body temperature is regulated at <10°C, but is generally ~2°C warmer than the ambient temperature when ambient temperature is >0°C. When ambient temperature falls below 0°C, central nervous system (CNS) signals activate thermogenic mechanisms and body temperature remains regulated near ~1°C (Richter et al., 2015). During entrance, torpor, and arousal, significant changes in neuronal activity occur. These changes have been evaluated by *in vivo* and *in vitro* electrophysiology as well as by documentation of c-Fos expression profiles and glucose utilization rate. Firing rates decrease; action potentials become smaller and broader with decreasing temperature during entrance into torpor; and they fully recover with increasing body temperature during arousal. Below 15–18°C, spontaneous activity ceases *in vivo*, but sensory stimulation leads to detectable responses in the CNS, even below 10°C body temperature. Accordingly, *in vitro* electrophysiology has shown: (i) cessation of spontaneous and electrically evoked activity at <5–16.6°C; and (ii) loss of LTP in the hippocampus at <15°C. The temperature-dependent changes in neuronal activity could be confirmed by equivalent changes in the expression of the activity marker c-Fos and reduced levels of glucose utilization. Abbreviations: AP, action potential; EEG, electroencephalogram; LTP, long-term potentiation; MPOA, medial preoptic area; SCN, suprachiasmatic nucleus.

Taken together, *in vivo* studies (Figure 1) convincingly show that although neuronal activity is attenuated throughout the brain in torpor, some brain regions are more active than others. These data led to the hypothesis that the limbic system-midbrain circuitry (interconnections between the limbic system, hypothalamus and brainstem reticular formation) contributes to neural control of entrance into and arousal from torpor (Heller, 1979; Beckman and Stanton, 1982).

## Electrophysiological Characterization of Neuronal Activity in the Hibernating Brain *in vitro*

Over the past 20 years, there has been a shift from electrophysiological *in vivo* studies to *in* *vitro* studies. *In vitro* approaches (brain slices) have the advantage that cellular mechanisms of neuronal activity in specific types of neurons can be more easily studied. Horowitz and colleagues (Hooper et al., 1985; Eckerman et al., 1990; Horrigan and Horowitz, 1990; Krelstein and Horowitz, 1990; Krelstein et al., 1990; Horowitz and Horwitz, 2019) and Igelmund and colleagues (Igelmund and Heinemann, 1995; Igelmund, 1995; Spangenberger et al., 1995) were pioneers investigating the temperature-dependent properties of CA1 hippocampal pyramidal neurons in brain slices. This early work included the finding that generation of long-term potentiation (LTP) in hamster brain slices is strongly dependent on temperature: i.e., it can be readily elicited above 20°C but not below 15°C (e.g., Krelstein et al., 1990). Similar observations were made for hippocampal long-term depression (LTD; Arant et al., 2011), leading to the view that neuroplasticity mechanisms contribute to hippocampal memory formation in euthermic but not in hibernating hamsters. That is, hamster LTP and LTD generation in CA1 hippocampal pyramidal cells appear silenced at and below 15°C (see Bronson et al., 2006; Hamilton et al., 2017) albeit CA1 hippocampal pyramidal cells continue to respond to afferent signals and generate action potentials at low slice temperatures.

Notably, at low slice temperatures, excitability of CA1 pyramidal neurons can still be increased by serotonin (Horrigan and Horowitz, 1990) and histamine (Nikmanesh et al., 1996; Hamilton et al., 2017). These studies implied that neuromodulators increased firing rates in specific classes of neurons so they could more effectively contribute to signal processing in torpor. In addition, *in vivo* histamine infusion into hippocampi of hibernating ground squirrels prolonged hibernation bouts (Sallmen et al., 2003b). Thus, *in vivo* studies and *in vitro* slice studies imply that histamine is an effective neuromodulator in hippocampal pyramidal neurons at low temperatures. Further support of a role for histamine in torpor was the observation of increased expression of histamine H1 and H2 receptors in the hippocampi of hibernating compared to euthermic ground squirrels (Sallmen et al., 2003a). Taken together, these data indicate that histaminergic modulation affects limbic system-midbrain circuitry in torpor. Specifically, histamine enhancement of CA1 pyramidal cell excitability may lead to inhibition of neurons in the brainstem reticular formation and prolongation of torpor bouts (Sallmen et al., 2003a; Hamilton et al., 2017).

Hibernating species have adaptations that greatly increase their tolerance to glucose and oxygen deprivation as first reported in the major study by Frerichs and Hallenbeck (1998), fully confirmed by Drew and colleagues (e.g., Ross et al., 2006), and reflected in sustained hamster hippocampal neural activity (Mikhailova et al., 2016). Still, it needs to be considered that ketones are preferred as energy supply in the brain during torpor (Krilowicz, 1985; Andrews et al., 2009). In addition, hippocampal slices of hibernating ground squirrels after 24 h in culture were shown to be more resistant to high doses of NMDA and KCl in comparison to slices of euthermic and non-hibernating animals (Ross et al., 2006). Also, inhibition of Na^+^/K^+^ pump did not lead to increased cell death in hippocampal slices of hibernating animals (Ross et al., 2006). It was therefore proposed that ion flux and the activity of the Na^+^/K^+^ pump is highly reduced during hibernation, leading to channel arrest and saving of energy. More recent data, however, indicate that in primary neurons of the somatosensory system (dorsal root ganglion), ion gradients, and membrane potential remain stable at 10°C in torpid animals (Hoffstaetter et al., 2018). Furthermore, a modification of NMDA receptor function was discussed to prevent neurons from tonic depolarization and exocytotoxic levels of intracellular calcium, which is presumed to be a consequence of inhibition of Na^+^/K^+^ pump and decrease of Na^+^ gradients (Ross et al., 2006; Zhao et al., 2006). In this context, it was proposed that the ion composition of the cerebrospinal fluid might change during hibernation to facilitate the depression of neuronal and synaptic activity (Igelmund and Heinemann, 1995; Igelmund, 1995).

Although most of the earlier and more recent *in vitro* studies focused only on the hippocampus, the influence of hypothermia in a hibernating species was also investigated in neurons of the medial preoptic area (MPOA), the suprachiasmatic nucleus (SCN) and the nucleus tractus solitarius (NTS), all of which are brain regions potentially being involved in neuronal control of hibernation. The MPOA controls thermoregulation and was considered to be especially important for the entrance into hibernation (Satinoff, 1967; Hashimoto et al., 1998). Consistent with the results obtained in the hippocampus, spontaneous neuronal activity of MPOA neurons recorded extracellularly in hamster brain slices significantly changed with decreasing temperature, resulting in reduced firing rate and spike amplitude as well as increased spike width (Hashimoto et al., 1998). Almost half of the recorded neurons (42%) were still active below 10°C with an average cutoff temperature of 4.9°C. Neurons of the rat MPOA exhibited similar results with 40% of neurons being active below 10°C, though the average cutoff temperature of 7.9°C was significantly higher compared to hamsters (Hashimoto et al., 1998). Extracellularly recorded spontaneous activity in the SCN also revealed temperature-dependent changes of neuronal activity. The majority of neurons were warm-sensitive, i.e., their firing rate decreased with decreasing temperature and the minimum temperature at which activity could be observed was 16.6°C (Miller et al., 1994). The NTS is located in the brainstem and receives peripheral input from the cardiovascular and pulmonary systems. Its 2nd order neurons support cardiovascular and respiratory reflexes (Taylor et al., 1999). Whole-cell patch-clamp recordings showed that these neurons generate less spikes with smaller amplitude and increased spike width after current injection at 15°C compared to 33°C. Similar observations were made for synaptically-driven activity (miniature and evoked EPSCs; Sekizawa et al., 2012). A very recent precise study of Hoffstaetter et al. (2018) presented whole-cell patch-clamp recordings of dorsal root ganglion neurons isolated from torpid squirrels, suggesting that the neuronal activity of these primary neurons of the somatosensory system is less sensitive to temperature-dependent changes than are neurons isolated from active squirrels. These results clearly demonstrate that action potentials could be generated through current injection at 10°C, though firing pattern changed from tonic to irregular or single spiking. The authors also showed that the membrane potential and input resistance was not affected at 10°C suggesting that ionic gradients and electric potentials are maintained in the dorsal root ganglion during hibernation.

Taken together, *in vitro* experiments are consistent with the *in vivo* experiments cited in the preceding section indicating that neural activity is markedly decreased in torpor. They further suggest that within a single neuron, some cellular mechanisms may be silenced in torpor (e.g., LTP and LTD), that firing rates in one type of neuron may decrease more rapidly than in other types within a specific nucleus (e.g., the NTS), and that neurons have protective adaptations sustaining neural activity in torpor.

## C-Fos Gene and Protein Expression and Glucose Utilization as a Measure of Neuronal Activity

In the early 1990s, evaluation of gene and protein expression of c-Fos, a proto-oncogene and immediate early gene product that is expressed upon neuronal activation was introduced as a new, innovative approach for indirect assessment of brain activity (Hoffman et al., 1993). Northern analysis or *in situ* hybridization of c-Fos RNA as well as immunhistochemical localization of c-Fos protein in brain slices have been proven as suitable tools to analyze neuronal activity. C-Fos RNA levels estimated by Northern analysis were reported to undergo dramatic changes throughout the hibernation cycle in virtually all parts of the brain, including hypothalamus, thalamus, cortex, midbrain, pons and medulla. In general, in euthermic animals and in early torpor, c-Fos RNA levels are low, slightly increase during deep torpor, peak in early arousal, and finally drop to low levels again in late arousal animals (Bitting et al., 1994; O’Hara et al., 1997, 1999). C-Fos *in situ* hybridization in coronal brain sections of ground squirrels helped to further specify these observations by neuroanatomical identification of relevant brain regions. Strong changes in c-Fos mRNA levels during the different phases of hibernation were found in the SCN, in the MPOA, and in the reticular thalamic nucleus (Bitting et al., 1994; Bratincsák et al., 2007). In the SCN and the MPOA, c-Fos mRNA changes are in accordance with the temporal profile of c-Fos expression revealed by Northern Analysis, confirming low levels of c-Fos expression during early torpor. In contrast, the reticular thalamic nucleus, which controls the communication between thalamus and cortex, exhibited an opposed profile with high c-Fos mRNA expression in the torpor state (Bratincsák et al., 2007). This is thought to be linked to inhibition of cortical activity supporting maintenance of torpor. Although this view is supported by low c-Fos levels (Bratincsák et al., 2007), there is increasing evidence that c-Fos might not be a reliable marker for neuronal activity during hibernation because transcription of genes and protein synthesis are profoundly depressed in deep torpor (van Breukelen and Martin, 2001, 2002).

A more direct approach to assess neuronal activity during hibernation is estimation of glucose metabolism. The glucose utilization rate in deep torpor is reduced to 1%–2% of the values of active animals (Frerichs et al., 1995). These data clearly confirm that neuronal activity is low in torpor, although, there are regional variations in the change of glucose uptake in the brain across the hibernation bout (Kilduff et al., 1990).

## Technical Limitations in Assessment of Neuronal Activity During Hibernation

While in *in vivo* studies, the physiological state of the hibernating brain is assumed to be preserved (except in the case of anesthesia usage), *in vitro* protocols only approximately simulate the physiological state which is—at the current state of knowledge—far from being standardized. One critical point is the preservation of brain temperature during all steps of preparation and handling of slices prior to recording (Pakhotin et al., 1993). Another point is that brains were often isolated from aroused/active hibernators with *hypothermia* data collected during exposure of brain slices to cold temperatures. There might be substantial differences when performing the same experiment in brains isolated from torpid animals (Hoffstaetter et al., 2018). Also, the composition of ACSF seems to be critical, because it may change across hibernation cycle (Pakhotin et al., 1993; Igelmund, 1995). Still, *in vitro* electrophysiology is indispensable for identifying the mechanisms underlying neuronal excitability in the hibernating brain. A comparable insight into cellular processes can hardly be achieved with common *in vivo* electrophysiological recording techniques (or indirect techniques). To compensate for hibernation-induced limitations in standard *in vitro* electrophysiology, a future alternative might be *in vivo* intracellular single-unit recordings in unanesthetized hibernators chronically implanted with specific recording devices that have been successfully conducted in rodents (Lee et al., 2006, 2009; Petersen, 2017).

## Summary and Concluding Remarks

The various approaches that have been used to characterize neuronal activity in the hibernating brain agree in one aspect: brain activity dramatically declines with decreasing body temperature (Figure 1). Neurons draw on glucose to support neuronal activity and in torpor, CNS glucose utilization has been lowered to 1%–2% of that of active animals. Still, the putatively most intriguing question (precisely what percentage of CNS neurons in various brain regions essentially suspend operating and seldom generate few action potentials in deep torpor?) evades a direct answer due to limitations of currently available experimental preparations and techniques. Present data imply that a low level of activity is maintained even in deep torpor, with many neurons generating action potentials infrequently. Interestingly, a variety of observations, from 2-deoxyglucose staining to single-unit recording indicate that neural activity varies in different brain regions and cell types. Moreover, in the hypothalamus and brainstem, neuronal activity, while low, maintains homeostatic temperature and cardiorespiratory regulation and responsiveness to alarm signals. For example, in deep torpor, a slow decline in ambient temperature to −30°C evokes a steady increase in activation of brown adipose tissue thermogenesis, keeping body temperature at ~1°C in Arctic ground squirrels (Richter et al., 2015) whereas a rapid fall in ambient temperature (an alarm signal) arouses the squirrel from hibernation. CNS regulation of the cardiorespiratory system ensures that all cells throughout the body receive oxygen, but not at a rate that would waste energy and defeat the entire advantage of hibernation. Taken together, despite a fundamental constraint on central signal processing in deep torpor (low brain temperature, Figure 1), evolutionary adaptations have configured critical subcortical signal processing (Table 1) to dependably support CNS function in a cold environment where food is scarce.

**Table 1:** Critical subcortical signal processing is preserved in deep torpor.

Brain region	Signal processing in deep torpor	Findings	References
Neocortex	Likely minimal if any	EEG activity (synchronous activity of neurons) is markedly attenuated.	Mihailovic (1972) and Heller (1979)
		Cognitive functions (learning, complex decision making, etc.) appear unneeded and largely suspended.	Strumwasser (1959)
Cerebellum	Likely minimal if any	Smooth, rapid coordination of muscle movements and execution of complex motor programs appear unneeded and suspended.	Strumwasser (1959)
Hypothalamus/suprachiasmatic nucleus	Likely minimal	Neuronal activity in the suprachiasmatic nucleus is markedly attenuated. Clock function is inhibited during deep torpor in arctic ground squirrels.	Miller et al. (1994); Ikeno et al. (2017) and Williams et al. (2017)
Hippocampus	Modulation of bout duration	EEG activity (synchronous activity of neurons) is attenuated.	Mihailovic (1972)
		Nonetheless, *in vivo* histamine infusion prolongs bout duration.	Sallmen et al. (2003a); Sallmen et al. (2003b)
Hypothalamus/Brainstem	Regulation of body and brain temperature	Thermosensitive hypothalamic/brainstem pathways regulating brain and core temperature remain active in deep torpor.	Florant and Heller (1977) and Heller (1979)
		One pathway activates brown adipose thermogenesis *via* sympathetic nerves. As amibient temperature is slowly lowered to −30°C, Arctic ground squirrels in torpor regulate body temperature at ~1°C by activating brown fat thermogenesis.	Morrison et al. (2014) and Richter et al. (2015)
Brainstem	Regulation of cardio-respiratory function	The fundamental brainstem respiratory rhythm generator remains functional in torpor. In addition, the baroreflex pathway supports blood pressure regulation. As a result, oxygen delivery to body cells is regulated so they remain viable throughout torpor.	Webb and Milsom (2017) and Horwitz et al. (2013)
	Spontaneous and alarm signal-driven arousals	Rapid decreases in ambient temperature and loud noises arouse the animal. Bouts are also naturally terminated (spontaneous arousals) in absence of alarm signals. In both cases, arousal appears to be mediated *via* the reticular arousal system.	Strumwasser (1959) and Beckman et al. (1976)

*The brain regions are color coded to indicate minimal (gray), modulatory (light orange) and essential (dark orange) signal processing in small mammalian species in torpor. The cited references provide data supporting the view that the CNS continues to process information and maintain homeostasis in torpor, control bout duration, and arouse the animal*.

## Author Contributions

Both authors have made a substantial, direct and intellectual contribution to the work, and approved it for publication.

## Conflict of Interest Statement

The authors declare that the research was conducted in the absence of any commercial or financial relationships that could be construed as a potential conflict of interest.
